# Ethical disclosure of biomarkers for Alzheimer risk in Latin American participants

**DOI:** 10.3389/frdem.2025.1672075

**Published:** 2025-10-28

**Authors:** Diana C. Oviedo, Rasheda Haughbrook, Carli Culjat, Ladanya Ramirez Surmeier, Adam E. Tratner, María B. Carreira, Alcibiades E. Villarreal, Sherelle L. Harmon, Oriana I. Batista, Zhuo Meng, Eugenia Millender, Casey D. Xavier Hall, Gabrielle B. Britton

**Affiliations:** ^1^Center for Neuroscience, Instituto de Investigaciones Científicas y Servicios de Alta Tecnología (INDICASAT-AIP), Panama City, Panama; ^2^Escuela de Psicología, Universidad Santa María La Antigua (USMA), Panama City, Panama; ^3^Sistema Nacional de Investigación (SNI) of the Secretaría Nacional de Ciencia Tecnología e Innovación (SENACYT), Panama City, Panama; ^4^Center of Population Sciences for Health Empowerment, College of Nursing, Florida State University, Tallahassee, FL, United States; ^5^Department of Psychology, Florida State University, Tallahassee, FL, United States; ^6^College of Social Sciences and Public Policy, Florida State University, Tallahassee, FL, United States; ^7^Pepper Institute on Aging and Public Policy, Florida State University, Tallahassee, FL, United States; ^8^Public Health Program, Florida State University, Tallahassee, FL, United States; ^9^Florida State University, Panama City, Panama; ^10^Center for Translational Behavioral Science, Florida State University, Tallahassee, FL, United States; ^11^Centro Gendiagnostik, S.A., Chiriquí, Panama; ^12^College of Social Work, Florida State University, Tallahassee, FL, United States; ^13^Centro de Vacunación e Investigación (CEVAXIN), Panama City, Panama

**Keywords:** Alzheimer’s disease, aging, Latin America, biomarkers, disclosure

## Abstract

**Introduction:**

In recent years, the disclosure of Alzheimer’s disease (AD) biomarkers has become increasingly common, offering critical insights into disease risk and progression. However, in low-resource settings, where healthcare access, provider training, and patient support are often limited, disclosing AD biomarkers presents unique ethical, logistical, and psychological challenges.

**Objective:**

This perspective explores the implications of AD biomarker disclosure in these settings, highlighting the potential risks of patient distress, misinformation, and inadequate follow-up care. For this purpose, we conducted a review of available literature, peer-reviewed studies, regional reports, and policy documents addressing AD in Latin America. Our literature search prioritized diagnostic advances, biomarker disclosure, treatment access, and health system challenges, providing a focused evidence base to frame the discussion of regional gaps and opportunities.

**Discussion:**

We discuss strategies to support responsible disclosure practices, including culturally sensitive participant education, enhanced provider training, and policy adaptations to improve accessibility and support systems. Ultimately, we advocate for a careful, context-specific approach to AD biomarker disclosure that prioritizes patient well-being and equity in low-resource environments.

## 1 Introduction

Innovation in biomarkers for Alzheimer’s disease (AD) has emerged largely through longitudinal research, providing critical insights into disease risk and progression. Given the implications of biomarkers for participants’ health and quality of life, the use of biomarkers in research raises important ethical questions regarding disclosure, including: (1) the ethical obligation to disclose, (2) the approach to disclosure, particularly in communicating biomarker limitations, (3) the psychosocial consequences of disclosure, and (4) the influence of context on ethical disclosure, especially in low-resource settings and minoritized communities. As these tools are refined, ethical and practical challenges surrounding biomarker disclosure and its implications for individuals must be carefully managed. Balancing scientific advancements with these considerations will be key to fully realizing the potential of biomarkers in clinical practice.

This perspective article examines the current state of AD biomarker disclosure in research settings, with a focus on low-and-middle income countries. It highlights the challenges faced in Latin American contexts regarding research and diagnosis of AD, as well as the limited evidence on practices and outcomes related to result disclosure. We conducted a focused literature review to inform this perspective on AD research and care in Latin America. Searches were carried out in PubMed, Scopus, and regional databases such as SciELO, using keywords including “Alzheimer’s disease,” “biomarkers,” “biomarker disclosure,” “Latin America.” We prioritized peer-reviewed publications from the last decade, as well as landmark studies, clinical guidelines, and policy reports from international organizations (e.g., World Health Organization, Alzheimer’s Association). Selection was based on relevance to diagnostic advances, treatment implementation, and health system disparities rather than comprehensive coverage. The literature reviewed provides the foundation for our critical analysis of regional challenges and opportunities to improve AD detection and care.

## 2 A brief perspective on the current state of research in Alzheimer’s disease biomarkers: relevance and disclosure

AD progresses over decades, from preclinical to dementia stages. Advances in biomarker research have shifted diagnosis toward a clinical-biomarker model (Tyagi et al., 2024). In 2024, the Alzheimer’s Association updated its criteria to emphasize biologically based diagnosis (Jack et al., 2024), incorporating amyloid-*β* and tau detection via PET and Cerebrospinal fluid (CSF). Since pathology precedes symptoms by years, biomarkers are essential for identifying at-risk individuals, including those with subjective complaints.

Blood-based biomarkers have emerged as accessible and scalable tools, though validation remains ongoing, particularly for early diagnosis (Hampel et al., 2023). Updated AD criteria now incorporate plasma markers (Jack et al., 2024), promoting integration with PET and CSF to improve staging (Hampel et al., 2023). Moreover, in May 2025, the Food and Drug Administration (FDA) approved the first blood test (Lumipulse G pTau217/ß-Amyloid 1–42 Plasma Ratio) that detects amyloid pathology linked to Alzheimer’s in cognitively impaired patients (FDA, 2025). Although research shows that biomarkers can signal AD neuropathology even in asymptomatic individuals and great advances have been made in diagnostics, disclosure remains ethically complex raising distress and uncertainty (Frisoni and Hansson, 2016; Gomez-Isla and Frosch, 2019). Moreover, given the limited availability of effective treatments, biomarker positivity may not directly translate into clinical benefit, although it can still support planning and decision-making.

Genetics also play a critical role: APOE ε4 is the strongest risk factor for sporadic late-onset AD, with homozygosity conferring particularly high risk (Farrer, 1997; Fortea et al., 2024). In low- and middle-income countries, genetic counseling must clarify differences between risk and diagnosis, explain testing limitations, and consider familial implications (Wouters et al., 2016; Hallquist et al., 2021). Beyond amyloid PET scans, Magnetic Resonance Imaging (MRI) provides valuable diagnostic information, distinguishing AD from other dementias and revealing atrophy in the medial temporal lobe and hippocampus in early disease (Živanović et al., 2022). However, disclosure practices vary widely: while U. S. studies show patients generally want and accept imaging results (Shoemaker et al., 2016), many low-resource settings lack standardized procedures (Vander Wyst et al., 2021; Erickson et al., 2024). Lastly, although blood-based markers can approach CSF accuracy, their stand-alone diagnostic value remains limited (Chen et al., 2021; Hardy-Sosa et al., 2022). Their predictive validity improves when combined with genetic, demographic, neuropsychological, and imaging data. Communicating such probabilistic risk poses ethical and psychological challenges, particularly in research with at-risk but asymptomatic individuals.

## 3 AD research in Latin America

Latin America faces a rapidly rising burden of AD, yet research, infrastructure, and therapeutic access remain limited. Regional populations are underrepresented in clinical trials, despite distinct risk profiles and social determinants that may shape disease presentation and treatment response (Llibre-Guerra et al., 2023). Barriers include limited funding and research infrastructure, scarcity of culturally and linguistically concordant teams, and logistical challenges that disproportionately affect Hispanic/Latino communities across the region constraining evidence tailored to local populations and health systems (Sosa et al., 2024). This underrepresentation is especially consequential as risk profiles and social determinants differ from highly studied cohorts in North America and Europe, yet these differences are rarely powered for subgroup analyses in pivotal trials (Llibre-Guerra et al., 2023).

Although the FDA approved lecanemab (Alzheimer’s Disease International, 2023) and donanemab (Alzheimer’s Disease International, 2024) access in Latin America lags by nearly five years on average due to regulatory delays (FIFAFIRMA, 2024). Mexico’s recent approval of lecanemab is an exception, but MRI surveillance and specialist care remain scarce in most public health systems (Barbosa et al., 2024). While treatments are slow to arrive, diagnostics are advancing. The 2024 Alzheimer’s Association criteria formally incorporate blood-based biomarkers, which offer scalable and cost-effective alternatives to PET and CSF in resource-limited settings (Jack et al., 2024). Early studies in Peru, Colombia, and Brazil show plasma p-tau217 and related markers perform well, though local validation is still needed (Barbosa et al., 2024). Moreover, in 2023, a review was published regarding a task force for diagnosis and treatment for people with AD in Latin America (Lopera et al., 2023). This review included multiple aspects that are relevant for diagnosis such as assessment recommendations, proposals for increasing training for primary care providers, developing region-specific or culturally adapted cognitive tests, expanding public healthcare coverage for testing and treatment, and implementing targeted search strategies for gene variants linked to AD. This underrepresentation is particularly consequential because risk profiles and social determinants differ from those of the highly studied cohorts in North America and Europe, yet these differences are seldom accounted for in subgroup analyses of pivotal trials. Nevertheless, although the report underscores the importance of biomarkers in research and clinical contexts, as other important reports on AD in the region, it does not explicitly address the ethical or practical aspects of biomarker disclosure to patients, highlighting the importance of its inclusion and examination. In line with this, the scarcity of studies from Latin America means they are often excluded from reviews; for example, a recent review on communicating AD-related risk did not include any studies conducted in the region (Swirska et al., 2025).

Although Latin America is faced with multiple challenges, progress in blood-based diagnostics provides a promising pathway for earlier detection and treatment readiness. Therefore, there is a growing need of studying the best possible way to disclose results. A coordinated investment in research capacity, biomarker validation, and health system preparedness are essential to ensure equitable access to emerging therapies(Schindler et al., 2024; Sosa et al., 2024).

## 4 Ethical, practical and psychosocial considerations and best practices in informing participants in low resource settings

### 4.1 Ethical considerations

Informing research participants about their individual test outcomes is a critical ethical obligation and should be rooted in the principles of respect for persons, beneficence, and justice. Respecting autonomy refers to considering participant’s self-determination, meaning individuals are free to decide whether to receive results (National Academies of Sciences, Engineering, and Medicine, 2018). Respect also requires transparent communication regarding findings that may impact their health, well-being, or medical decisions. Moreover, the principle of beneficence requires researchers to ensure participants’ well-being, reducing potential harms and risks. The National Institutes of Health (NIH) and World Health Organization (WHO) emphasize the importance of informing participants about new information that might emerge during research, which could change their assessment of the risks and benefits of participating in the research (National Institutes of Health, 2021; WHO, 2024). The information not only influences participants’ decisions about continuing their involvement but also their personal health choices. Challenges of participant data release include both scientific and ethical dilemmas including determining which results to share, the actionability of the findings, logistic complexities of delivering accurate and comprehensible communication, and financial considerations for both researcher and participant. The transparency of timely and clear communication about results to participants fosters trust and upholds the ethical integrity of the research process. Furthermore, the ethical principle of justice implies all participants must be treated equally and must be included in the different stages of the study. Regarding biomarker disclosure, all individuals must be given the same opportunities to receive results.

### 4.2 Best practices for disclosure

Implementing best practices for returning results involves several key strategies. First, researchers should ensure that the information conveyed to a participant is clear and comprehensible, tailoring all the information to a participant including literacy levels, communication preferences, and cultural context, as meaning may differ in cross-cultural settings (Rojas-Guyler et al., 2016; Zegers and Auron, 2022). Participants should understand all potential outcomes, risks, and implications of the biomarker testing/results, including what is still unknown. This promotes responsible transparency, autonomy, informed decision-making, and helps build community trust (Zegers and Auron, 2022). Communicating biomarker results in low-and-middle income countries faces some challenges like the lack of genetic counselors and bioinformaticians. Comprehensive genetic counseling protocols are available for AD diagnostic and predictive testing to provide a framework to evaluate which patients may benefit from genetic testing (Goldman et al., 2011). Bioinformaticians are essential as partners to molecular geneticists and are fundamental to collect and help identify new genetic variants that may improve diagnostic capabilities or counseling of risk for the patient.

An example of this is the NIH *All of Us* Research Program which illustrates ethical disclosure by returning personalized DNA results with clear, accessible reports on health risks. Participants receive education on risks and benefits before data collection and, upon disclosure, are provided resources such as free genetic counseling to help interpret results accurately (Sankar and Parker, 2017). Similarly, U. S. policy now mandates open access to patient data through the 2021 *OpenNotes* legislation, which requires immediate availability of lab findings, radiology reports, and clinician notes via patient portals. Studies show patients prefer this approach (Steitz et al., 2023).

Adopting principles of transparency and intentional disclosure can improve communication of test results, research findings, and recommendations, thereby enhancing engagement and informed decision-making. To address ethical challenges, researchers should develop biomarker disclosure guidelines that account for genetic and environmental contexts, integrate disclosure into protocols and consent, and involve IRBs in establishing best practices.

Continuous support and counseling are essential for participants receiving biomarker results. This includes expanded access to care, referrals to community resources, and the use of telehealth to improve counseling availability (Boothe et al., 2021). Providing guidance on lifestyle changes that may lower AD risk, even for those with high genetic susceptibility, can further empower participants to take preventive action (Vernarelli et al., 2010).

It is also essential to recognize the structural inequities that may limit access to care, particularly for historically marginalized communities, and advocate for public health interventions that address these disparities. Currently, some recommendations include setting participant expectations, creating materials with guidance by end-users in primary languages and considering cultural implications, using plain language, and using accessible resources to facilitate understanding (National Academies of Sciences, Engineering, and Medicine, 2018). A growing body of literature highlights the benefits of involving key community stakeholders, such as patients, family caregivers, and healthcare professionals, throughout the research process (Alpinar-Sencan and Schicktanz, 2020). Engaging stakeholders in biomedical research ensures that the needs of those most affected are addressed. Frameworks such as Co-Design promote systematic collaboration with end-users, leading to more relevant and effective interventions (Bird et al., 2021; Bloska et al., 2024). Stakeholder involvement also deepens understanding of lived experiences, improves enrollment and retention, and enhances data quality and rigor. From a patient-centered perspective, incorporating participant voices affirms autonomy and supports ethical research practices (Beier et al., 2019).

Lastly, peer support and education are also effective among both care providers and caregivers of people with AD or other forms of dementia. Care providers who participated in training with patients and patient families as peer educators gained a better understanding of the complexity of living with dementia and were able to adjust their delivery of care (Jack-Waugh, 2023).

Among those who serve as caregivers, having peer support reminded them that there are others who are also experiencing the same challenges and gave them the space to share their experiences (Greenwood et al., 2013). In addition, support helplines and multiple versions of online support systems have shown positive effects. Online support groups of peer caregivers help users understand dementia symptoms, provide emotional support and coping strategies, however, without proper training these online peers may not always be providing accurate information (Yin et al., 2024). While online support groups increase the accessibility of finding peers, some communities may have limited internet access. Furthermore, for those caregivers who are still looking for assistance on daily caregiving aspects or those who are “reluctant” caregivers require more defined peer support (Knight et al., 2024).

Peer support groups have long benefited individuals with AD by providing emotional support, coping strategies, practical resources, and reducing isolation, while enhancing self-esteem and self-efficacy (Coulson and Talbot, 2025). Online formats now expand these benefits, offering flexible, targeted spaces such as groups for young-onset dementia (Craig and Strivens, 2016) or prevention-focused communities encouraging physical activity. Peer-led interventions also foster resilience among those living with dementia (Whelan et al., 2020). However, gaps remain in communicating diagnostic results, where trained peer educators could help bridge providers and patients by ensuring timely, understandable information.

### 4.3 Psychosocial implications of biomarker disclosure

Considering the psychosocial impact of biomarker disclosure is fundamental for understanding and coping with biomarker test results in relation to prognosis. Previous research has identified several factors related to participants’ psychosocial response to biomarker disclosure, such as level of impairment, resilience, adaptation, and specific coping mechanisms (Vernarelli et al., 2010; Lineweaver et al., 2014; Green et al., 2015). Some work suggests that the ways in which disclosure is communicated (e.g., specific language used, clarity of the disclosure), the context in which disclosure takes place (e.g., at the clinic or home), the availability of social support, and the availability of information regarding future possible treatments may also influence individuals’ response to biomarker disclosure (Couch et al., 2024). For some individuals, disclosure can be a positive experience as it helps them better understand their condition and what to expect in the future, which can reduce uncertainty and provide a sense of control over the situation. People might feel more prepared to face their condition as receiving information can help them make better and more informed decisions about legal, financial, family and healthcare matters (Bemelmans et al., 2016). Moreover, in some contexts where there are ongoing clinical trials or intervention studies, individuals aware of their results, might be interested in volunteering as research participants (Grill et al., 2016).

Receiving results can also be associated with distress, anxiety, depression, uncertainty, fear, and a sense of helplessness, feeling like there is no hope left after a diagnosis. Likewise, disclosure can be associated with an increased risk of stigmatization (Ketchum et al., 2024). Individuals may fear being stigmatized or discriminated against due to their biomarker status, which could impact different aspects of their lives, such as health services availability, employment, or relationships. Disclosure can also affect family members, who may experience their own emotional reactions. In some cases, family members may struggle with how to support the person or may feel burdened by the information.

Some studies suggest that the distress associated with disclosure is higher among people with mild cognitive impairment (MCI). A systematic review exploring psychosocial and behavioral outcomes after amyloid PET scan disclosure found that impaired individuals with elevated amyloid levels experienced greater distress and anxiety, whereas for participants without MCI, biomarker disclosure was not associated with anxiety, depression, or suicidality (Bemelmans et al., 2016; Grill et al., 2016). Nevertheless, reviews (that do not include Latin American participants) report that anxiety and depression remain low (Swirska et al., 2025). This highlights the need to study the psychological impact in Hispanic populations. Biomarker disclosure can be an emotionally distressing experience even when the results are inconclusive. Researchers should communicate findings with clarity and empathy to minimize anxiety and confusion (Arvisais-Anhalt et al., 2023). The empathy shown by personnel is essential for ameliorating the psychosocial impact of biomarker disclosure (Couch et al., 2024). Furthermore, individualized counseling and psychoeducation to individuals and their families following disclosure may aid in understanding the implications of the results.

## 5 Discussion

As evidence grows, AD diagnostic criteria will continue to evolve, incorporating new blood-based biomarkers to improve accessibility, affordability, and early detection. These biomarkers promise to complement established CSF and PET measures while addressing the need for scalable diagnostic solutions. Disclosing AD biomarker information is necessary; however, it poses ethical and clinical challenges. Ethical disclosure entails acknowledging the diversity of human populations and should focus on identifying and informing individuals who have greater susceptibility to AD relative to other individuals within a population. Moreover, ethical disclosure should simultaneously steer individuals toward access to care that suits their medical and psychosocial needs, while considering their broader socioeconomic and cultural context. The delivery and interpretation of results should be personalized, with culturally sensitive communication that respects community autonomy and promotes informed decision-making. In Latin America, to our knowledge, no studies have incorporated biomarker disclosure in aging and dementia studies. The burden of AD is increasing rapidly in the region, yet research, infrastructure, and therapeutic access remain limited. Although research on diagnosis and treatments are advancing, ethical and practical aspects of biomarker disclosure remain largely absent from these discussions, and reviews on risk communication rarely include Latin American data (Swirska et al., 2025). Studies focusing on context-specific approach to AD biomarker disclosure that prioritizes patient well-being and equity in low-resource environments are essential for fostering trust, advancing health equity, and supporting disease prevention efforts in diverse communities as well as ensuring the ethical advancement of Alzheimer’s disease and Related Dementias (ADRD) research.

## Data Availability

The original contributions presented in the study are included in the article/supplementary material, further inquiries can be directed to the corresponding author/s.
